# Comparative evaluation of microneedling-assisted injectable PRP versus PRP for managing thin gingival phenotype: A randomized clinical study

**DOI:** 10.6026/973206300222869

**Published:** 2026-05-31

**Authors:** Pawan Kumar, Maya S Indurkar, Dhalkari C.D, Madhu Varma, Aishwarya Mulay

**Affiliations:** 1 Department of Periodontology, Government Dental College and Hospital, Chhatrapati Sambhajinagar, Maharashtra, India

**Keywords:** Microneedling, platelet-rich plasma (PRP), thin gingival phenotype, gingival thickness, periodontal regeneration

## Abstract

Thin gingival phenotype presents a clinical challenge due to its susceptibility to recession and compromised periodontal stability. Therefore, it is of interest to compare the effectiveness of microneedling-assisted injectable platelet-rich plasma (PRP) with PRP in managing thin gingival phenotype. Hence, patients were allocated into two groups receiving either combined microneedling with PRP or PRP and outcomes were assessed based on gingival thickness and clinical parameters. The combination therapy demonstrated significantly greater improvement in gingival thickness and overall tissue quality compared to PRP alone. Thus, we show that microneedling enhances the regenerative potential of PRP, making it a more effective modality for gingival phenotype modification.

## Background:

Modern periodontal therapy emphasizes achieving optimal aesthetic outcomes along with functional stability. The morphological characteristics of periodontal soft and hard tissues significantly influence treatment success and long-term prognosis. The concept of periodontal phenotype has been introduced to describe the dimensional relationship between gingival tissues and supporting alveolar bone. Gingival phenotype is primarily evaluated based on gingival thickness and keratinized tissue width, while bone morphotype is assessed by buccal bone plate thickness. These anatomical parameters play a vital role in determining the susceptibility to periodontal diseases, mucogingival deformities and outcomes of periodontal and implant therapy [1]. Individuals with thin gingival phenotype are more susceptible to gingival recession, mechanical trauma and compromised aesthetic results following periodontal or restorative interventions. Reduced gingival thickness has been associated with increased risk of tissue breakdown under inflammatory or traumatic conditions. In contrast, thick gingival phenotypes demonstrate greater resistance to recession but may show a tendency toward periodontal pocket formation. Consequently, enhancement of gingival thickness has gained considerable importance in modern periodontal treatment planning [2, 3]. Minimally invasive approaches aimed at increasing gingival thickness have gained popularity due to reduced patient morbidity and improved healing response. Microneedling, also known as percutaneous collagen induction therapy, involves controlled micro-injury of tissues which initiates a wound healing cascade. This process stimulates the release of growth factors such as platelet-derived growth factor, transforming growth factor-β and fibroblast growth factor. These biological mediators promote fibroblast proliferation, neocollagenesis, neoangiogenesis and subsequent tissue remodeling [4, 5].

Autologous platelet concentrates have also shown promising regenerative potential in periodontal therapy. Injectable platelet-rich plasma (I-PRP) is a liquid platelet concentrate obtained through centrifugation of autologous blood. It contains high concentrations of platelets, leukocytes and growth factors that enhance tissue repair and regeneration. The injectable nature of I-PRP allows better distribution within connective tissues and provides sustained release of growth factors that facilitate angiogenesis and collagen synthesis [6, 7, 8]. Although microneedling and platelet concentrates have shown beneficial effects individually, limited studies have evaluated their combined regenerative potential. Therefore, it is of interest to evaluate the combined effect of microneedling-assisted injectable platelet-rich plasma compared with platelet-rich plasma in improving gingival thickness and keratinized tissue width in individuals with thin gingival phenotype.

## Materials and Methods:

The study was conducted at government hospital in 2025 after the protocol had been approved by the Institutional Committee of Research Ethics. All the participants were informed about the importance of the study and written informed consent was taken from the patient. All patients with clinical diagnosis of thin gingival phenotype were considered eligible. Patients were clinically evaluated and recorded.

## Inclusion criteria:

[1] Systemically healthy patients aged 18-55 years

[2] Patients willing to undergo treatment with written informed consent

[3] Presence of thin gingival phenotype (gingival thickness <1.5 mm)

[4] Sites involving maxillary and mandibular canine and first premolar regions

[5] Good oral hygiene status 

## Exclusion criteria:

[1] Patients undergoing active orthodontic treatment

[2] Patients taking medications causing gingival enlargement or anticoagulant/blood thinners or systemic diseases

[3] Presence of pre-existing mucogingival deformities

[4] History of bruxism or parafunctional habits

[5] Previous history of periodontitis or periodontal surgery

[6] Pregnant or lactating women

[7] Use of tobacco in any form 

A total of 20 patients presenting with thin gingival phenotype were included in the study. Gingival phenotype was determined using a transgingival probing technique. An endodontic file was gently inserted perpendicularly into the gingival tissue under local anesthesia and the penetration depth was measured using an electronic digital caliper. Gingival thickness measuring less than 1.5 mm was categorized as thin gingival phenotype. The present study was designed as a split-mouth clinical study. In each patient, the test sites in the lower quadrant were treated with microneedling combined with injectable platelet-rich plasma (I-PRP), whereas the contralateral sites served as control sites and were treated with I-PRP alone. Following treatment, patients were recalled at 1 month and 3 months to evaluate changes in gingival thickness. Gingival thickness at follow-up visits was assessed using the same standardized transgingival probing method with an endodontic file and electronic digital caliper to ensure reproducibility and measurement accuracy. All clinical procedures were performed by a single experienced operator to minimize inter-operator variability and ensure consistency in treatment delivery and measurement techniques.

## Results:

**Figure 1:**
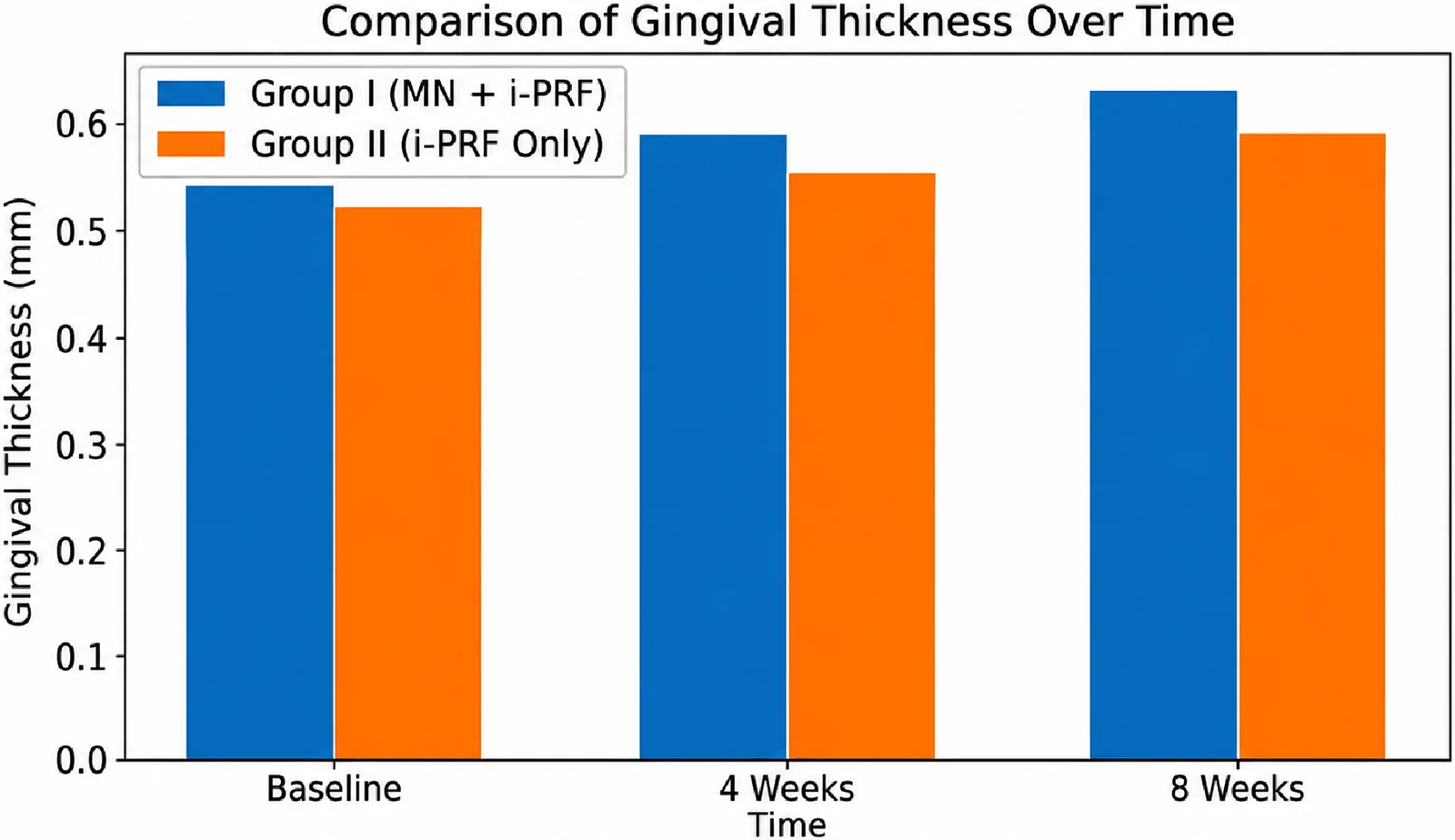
Results comparing at baseline, 4weeks and 8weeks

At baseline, gingival thickness between the test and control groups showed no statistically significant difference (p = 0.18), indicating homogeneity between the groups prior to intervention. At 1 month follow-up, both groups demonstrated an increase in gingival thickness; however, the increase observed in Group I (Microneedling + I-PRP) was statistically significant compared to Group II (p = 0.04). At 3 months follow-up, a further increase in gingival thickness was observed in both groups. Group I continued to demonstrate significantly greater gingival thickness compared to Group II (p = 0.02) (Table 1, Figure 1).

## Discussion:

The periodontal phenotype plays a significant role in determining the success and long-term stability of periodontal, implant and restorative treatments. Among the various components of periodontal phenotype, gingival thickness is considered a critical determinant influencing tissue resistance to trauma, inflammation and mucogingival deformities. Thin gingival phenotype is frequently associated with increased susceptibility to gingival recession and compromised aesthetic outcomes, thereby necessitating therapeutic interventions aimed at soft tissue augmentation [8]. In the present study, both treatment modalities demonstrated an improvement in gingival thickness over the evaluation period. However, the group treated with microneedling combined with injectable platelet-rich plasma demonstrated significantly greater enhancement in gingival thickness compared to the group treated with injectable platelet-rich plasma alone. The improved outcomes observed in the test group may be attributed to the synergistic biological effects of microneedling and platelet-derived growth factors. Microneedling produces controlled micro-injuries in the gingival tissue, initiating a cascade of wound healing responses characterized by increased fibroblast proliferation, collagen synthesis and neoangiogenesis. Previous studies have demonstrated that microneedling stimulates the release of multiple growth factors including platelet-derived growth factor and transforming growth factor, which promotes extracellular matrix formation and tissue remodeling [9, 1]. Enhanced vascularization and fibroblast activity following microneedling contribute to increased gingival thickness through neocollagenesis and tissue maturation. Injectable platelet-rich plasma has been widely recognized for its regenerative potential due to its high concentration of platelets, leukocytes and biologically active growth factors. These growth factors facilitate cellular migration, angiogenesis and collagen production, thereby accelerating softtissue healing. Additionally, the injectable nature of I-PRP allows uniform distribution within the connective tissue and provides sustained release of growth factors at the site of injection [6, 7, 11]. The combination of microneedling with I-PRP may further enhance tissue permeability, thereby improving the diffusion and bioavailability of growth factors within the gingival tissues. The findings of the present study are consistent with previous reports suggesting that gingival thickness greater than 1 mm is associated with improved clinical stability and reduced risk of post-treatment gingival recession [12]. Furthermore, studies evaluating mucogingival procedures have reported that thicker gingival tissues demonstrate improved root coverage outcomes and enhanced resistance to mechanical trauma [13]. These findings support the clinical relevance of gingival phenotype modification in contemporary periodontal therapy. The use of transgingival probing for gingival thickness measurement in the present study provided a reliable, reproducible and cost-effective method for clinical evaluation. A previous investigation has demonstrated that transgingival probing shows comparable accuracy to ultrasound measurements and remains a widely accepted clinical technique [14]. Multiple treatment sessions were performed in the present study at 10-day intervals. This interval was selected based on evidence suggesting that peak collagen synthesis occurs within 7-14 days during the wound healing process and growth factor release from platelet concentrates occurs within a similar time frame [15,16]. The repeated application of microneedling and I-PRP may therefore contribute to sustained stimulation of tissue regeneration. Despite the favorable outcomes observed, certain limitations were noted. The injection of I-PRP was restricted to the apical mucogingival region due to the dense structure of keratinized gingiva. Additionally, the relatively small sample size and short follow-up duration limit the generalizability of the findings. Further long-term randomized clinical trials with larger sample sizes are required to validate the effectiveness and stability of this minimally invasive technique. Overall, the results of the present study suggest that microneedling combined with injectable platelet-rich plasma may serve as a promising non-surgical approach for gingival phenotype modification, providing enhanced soft tissue augmentation and improved aesthetic outcomes.

## Conclusion:

Both injectable PRP and microneedling with PRP significantly improved gingival thickness in patients with thin gingival phenotype. The combination therapy showed superior outcomes, likely due to enhanced neocollagenesis, neoangiogenesis and improved growth factor delivery. Microneedling with PRP appears to be a promising minimally invasive approach.

## Figures and Tables

**Table 1:** Comparison of gingival thickness between groups

**Time Interval**	**Group I (Microneedling + I-PRP) Mean ± SD (mm)**	**Group II (I-PRP Alone) Mean ± SD (mm)**	**p-value**
Baseline	0.54 ± 0.03	0.52 ± 0.03	0.18 (NS)
1 Month	0.59 ± 0.03	0.55 ± 0.03	0.04*
3 Months	0.63 ± 0.03	0.59 ± 0.03	0.02*
*Statistically
significant (p < 0.05)
NS-Not statistically
significant
